# Impact of Prehospital Care on Outcomes in Sepsis: A Systematic Review

**DOI:** 10.5811/westjem.2016.5.30172

**Published:** 2016-07-05

**Authors:** Michael A Smyth, Samantha J Brace-McDonnell, Gavin D Perkins

**Affiliations:** University of Warwick, Clinical Trials Unit, Coventry, England

## Abstract

**Introduction:**

Sepsis is a common and potentially life-threatening response to an infection. International treatment guidelines for sepsis advocate that treatment be initiated at the earliest possible opportunity. It is not yet clear if very early intervention by ambulance clinicians prior to arrival at hospital leads to improved clinical outcomes among sepsis patients.

**Methoda:**

We systematically searched the electronic databases MEDLINE, EMBASE, CINAHL, the Cochrane Library and PubMed up to June 2015. In addition, subject experts were contacted. We adopted the GRADE (grading recommendations assessment, development and evaluation) methodology to conduct the review and follow PRISMA (Preferred Reporting Items for Systematic Reviews and Meta-Analyses) recommendations to report findings.

**Results:**

Nine studies met the eligibility criteria – one study was a randomized controlled trial while the remaining studies were observational in nature. There was considerable variation in the methodological approaches adopted and outcome measures reported across the studies. Because of these differences, the studies did not answer a unique research question and meta-analysis was not appropriate. A narrative approach to data synthesis was adopted.

**Conclusion:**

There is little robust evidence addressing the impact of prehospital interventions on outcomes in sepsis. That which is available is of low quality and indicates that prehospital interventions have limited impact on outcomes in sepsis beyond improving process outcomes and expediting the patient’s passage through the emergency care pathway. Evidence indicating that prehospital antibiotic therapy and fluid resuscitation improve patient outcomes is currently lacking.

## INTRODUCTION

Sepsis is a common and potentially life-threatening response to an infection.^1^ There are an estimated 150,000 cases of severe sepsis resulting in more than 44,000 deaths each year in the United Kingdom (UK).^2^ It has been reported that over 70% of sepsis cases stem from the community^3^ with one study suggesting two-thirds of severe sepsis cases are initially seen in the emergency department (ED).^2^ Approximately half of all ED sepsis patients will arrive via emergency medical services (EMS).^5^–^10^ Sepsis patients transported to the ED by EMS are likely to be sicker than those arriving by other means,^6^, ^8^–^11^ with up to 80% of severe sepsis patients admitted to intensive care from the ED having been transported by EMS.^7^,^12^

International treatment guidelines for sepsis advocate that treatment be initiated at the earliest possible opportunity.^1^ It has been argued that early intervention by ambulance clinicians prior to arrival at the ED may lead to improved outcomes among sepsis patients^13^ in the same manner as EMS intervention has helped to improve outcomes for other time critical, life-threatening conditions such as acute myocardial infarction^14^, stroke^15^, and major trauma.^16^

## METHODS

This systematic review addresses the impact of prehospital care on outcomes among patients with sepsis. The review adopted the Grading of Recommendations, Assessment, Development and Evaluation (GRADE) methodology^17^ and is reported consistent with the Preferred Reporting Items for Systematic Reviews and Meta-Analyses (PRISMA) recommendations.^18^

### Inclusion Criteria

Studies were eligible for inclusion if they reported the impact of prehospital care among adult patients with suspected sepsis (including severe sepsis and septic shock). Outcomes of interest include time to early goal-directed therapy (EGDT) related targets, admission to intensive care unit (ICU), length of stay and mortality. We included conference proceedings/meeting abstracts to capture gray literature.

### Search Strategy Electronic Searches

We systematically searched MEDLINE, EMBASE, CINAHL, the Cochrane Library and PubMed. No language restrictions were employed.

### Search Terms/Search Strategy

Search strategies were based upon the terms below: (Sepsis OR septic OR septic?emia OR systemic adj inflammatory adj response adj syndrome OR SIRS OR septic adj shock OR hypotension adj induced adj hypoperfusion OR cryptic adj shock OR bacterial adj infection) AND (emergency adj medical adj service OR EMS OR HEMS OR emergency adj medical adj technician OR EMT OR paramedic OR pre-hospital OR prehospital OR pre adj hospital OR out-of-hospital OR out adj of adj hospital OR OOH OR Ambulance).

The initial MEDLINE search was conducted in July 2014 and adapted for each subsequent database. The searches were repeated in June 2015 to identify recent publications.

### Other

We contacted subject experts and scrutinized reference lists of included manuscripts in order to identify any missed studies.

### Data Collection And Analysis Study Selection

Study selection occurred in two stages. First, two reviewers (MAS and SJBM) independently reviewed each citation and abstract against the inclusion criteria. Citations rated as ‘include’ by either reviewer were retained; citations rated as ‘exclude’ by both reviewers were rejected. Second, full manuscripts of retained citations were independently screened by two reviewers (MAS and SJBM) who rated each manuscript as ‘include,’ ‘maybe,’ or ‘exclude’ against the inclusion criteria. If both reviewers rated a manuscript as ‘include’ it was included for critical appraisal. If both reviewers rated a manuscript as ‘exclude’ it was automatically rejected. If the two reviewers had differing opinions, the reviewers discussed the manuscript in order to achieve consensus. If the reviewers were unable to agree following discussion, a third independent reviewer (GDP) was available to adjudicate.

### Risk Of Bias

For randomized controlled trials, we assessed risk of bias across the following domains: lack of allocation concealment, lack of blinding, incomplete accounting of patients and outcome events, selective outcome reporting bias and other limitations such as stopping a trial early for benefit. For observational studies, bias was assessed across the domains of failure to develop and apply appropriate eligibility criteria (inclusion of control population), flawed measurement of exposure and outcome, failure to adequately control confounding and incomplete follow up.

All papers were assessed across their respective domains with each being categorized as either high risk, low risk or level of risk unclear as per GRADE recommendations.^19^ We considered studies categorized as high risk in any domain to be at high risk of bias overall. Studies categorized as low risk across all domains were considered to be at low risk of bias overall. Studies with a combination of low and unclear risk across domains were considered to have an unclear risk of bias overall.

### Quality Of Evidence

We determined quality of evidence according to the GRADE framework. Study design informed initial quality presumptions; randomized controlled trials were initially presumed to be ‘high quality,’ while observational studies (non-randomized studies) were initially presumed to be ‘low quality.’ Two reviewers (MAS and SJBM) appraised each paper across the five core GRADE domains of risk of bias,^19^ inconsistency,^20^ indirectness,^21^ imprecision^22^ and other considerations (including publication bias).^23^ If any concerns were identified quality of evidence was adjusted downward. Similarly, quality could be adjusted upward if, for example, a large treatment effect or dose response was noted, that subsequently raised confidence in the estimate of effect.^24^ Ultimately each study is rated as follows:

- High quality: We are very confident that the true effect lies close to that of the estimate of effect.
- Moderate quality: We are moderately confident in the effect estimate: the true effect is likely to be close to the estimate of effect, but there is a possibility that it is substantially different.
- Low quality: Our confidence in the effect is limited: the true effect may be substantially different from the estimate of the effect.
- Very low quality: We have very little confidence in the effect estimate: the true effect is likely to be substantially different from the estimate of effect.

## RESULTS

### Study Inclusion

Database searches yielded 4,366 citations. Duplicate citations were removed manually within EndNote® (version X7 Thompson Scientific, Carlsbad, CA) by a single reviewer (MAS) providing 2,958 unique citations. One citation was identified by contacting subject experts. After the first stage of screening 79 citations were retained and 2,880 citations were rejected. Inter-rater agreement for first stage screening, calculated using Cohens kappa statistic, was 0.87 (95% CI [0.81 to 0.92]). During the second stage of screening 79 manuscripts were reviewed; 70 were discarded following assessment and nine were retained for critical appraisal (Figure). Inter-rater agreement for second stage screening, calculated using Cohens Kappa, was 0.88 (95% CI [0.72 to 1.0]).

No additional citations were identified by scrutinizing the reference lists of included manuscripts. One additional study,^25^ a manuscript pending publication (subsequently published), was identified by contacting subject experts. In total nine studies are included in the final analysis (Figure).

### Characteristics Of Included Studies

Characteristics of included studies, comprising 3,470 patients in total, are summarised in the Table.

### Risk Of Bias Findings

Risk of bias assessments are reported in Tables 2 and 3.

### Quality Of Evidence Findings

We identified very low quality evidence from one randomized controlled trial (downgraded for risk of bias, indirectness and imprecision), and very low quality evidence from eight observational studies (downgraded for risk of bias, indirectness and imprecision across studies, see supplementary information for evidence table with quality assessment.)

### Data Synthesis

There was considerable variation in the methodological approach adopted across the studies as well the outcome measures reported. The majority of studies identified involve limited numbers of participants, without comparable control and intervention cohorts. Because of these differences, the studies did not answer a unique research question thus meta-analysis was not appropriate. A narrative approach to data synthesis was adopted.

### Data Extraction

The data from included studies were extracted and entered into the evidence table (see Appendix A) and summary of findings table (Table 4) by a single reviewer (MAS) and verified by a second reviewer (SJBM).

## ANALYSIS

### Antibiotic Therapy

Three studies indicate that ED antibiotic therapy is administered 30–50 minutes sooner if EMS identify sepsis and inform the receiving clinician of their diagnosis.^5^,^11^,^26^ However, this finding is not universal – Guerra et al.^27^ failed to identify any significant reduction in time to antibiotic therapy (pre-alert: 72.6 minutes Standard Deviation (SD) 59.3 minutes) vs no pre-alert: 98.5 minutes (SD 89.9 minutes), p=0.07). None of the studies concerned with prehospital recognition of sepsis, without concomitant administration of antibiotics, were able to identify any significant improvement in length of stay^11^,^25^,^27^ or mortality.^11^,^25^–^28^

Two studies^29^,^30^ address prehospital administration of antibiotic therapy. Chamberlain^25^ reported that antibiotics were delivered 3.4+−2.6 hours sooner while Bayer *et al.*^30^ noted that among EMS sepsis patients median time to antibiotics was 19 minutes (IQR 18–24 minutes) from initial emergency call (time of administration was estimated to commence 10 minutes after arriving at scene). Bayer *et al.*^30^ do not report interval to hospital nor report time to antibiotics in the ED. Chamberlain^29^ suggests that prehospital antibiotic therapy leads to reduced intensive care unit (ICU) stay (Mean ICU stay: 6.8±2.1 days (intervention) vs 11.2±5.2 days (control), p=0.001) and reduced mortality (28-day mortality: 42.4% (intervention) vs 56.7% (control); odds ratio (OR) 0.56; 95% CI [0.32–1.00]). Bayer *et al.*^26^ did not report mortality, ICU admission or length-of-stay data.

### Intravascular Fluid Therapy

Band *et al..*^26^ reported that arrival by EMS reduces time to initiation of intravascular fluid therapy when compared with those who arrive by privately owned vehicle (POV, EMS: 34 minutes [IQR 10–88 minutes] vs POV: 68 minutes, IQR 25–121 minutes, p≤0.001), but did not improve mortality (adjusted risk ratio [RR] 1.24; 95% CI [0.92–1.66]). Similarly Bayer *et al.*^30^ noted that among EMS sepsis patients median time to initiation of Intravenous fluids was 19 minutes (IQR 18–24 minutes) from initial emergency call (time of administration was estimated to commence 10 minutes after arriving at scene), with patients receiving an average of 2.5l intravascular fluid (IQR 1.5–3.01) until admission to the ED. A third study by Guerra *et al.*^27^ indicated that early identification of sepsis by EMS was not associated with improved six-hour fluid resuscitation targets in the ED (EMS pre-alert: 42.97 cc/kg (SD 33.23cc/kg) vs no EMS pre-alert: 35.17cc/kg (SD 26.81 cc/kg, p=0.30).

The only study to demonstrate a positive impact following prehospital fluid administration among sepsis patients indicated that prehospital fluids were associated with reduced likelihood of organ failures (adjusted OR 0.58; 95% CI [0.34–0.98]) and reduced hospital mortality (adjusted OR 0.46; 95% CI [0.23–0.88]), but not reduced ICU admission (adjusted OR 0.64; 95% CI [0.37–1.10]).^31^ The median volume of prehospital fluid administered in this study was 500mL (IQR 200–1000mL).

### Early Goal Directed Therapy (EGDT) Targets

Femling *et al.*^11^ reported that patients who arrived at the ED via EMS had shorter time to central line placement (required for central venous pressure monitoring) than those who arrived by other means (EMS: 200 minutes [IQR 89–368 minutes] vs non-EMS: 275 minutes [IQR 122–470 minutes], difference 75 minutes, p<0.01), while Guerra *et al.*^27^ noted that when EMS provided a sepsis pre-alert to the hospital the advance notification it did not impact the decision to place a central venous catheter (EMS pre-alert: 61% vs no EMS pre-alert: 68%, p=0.54). Although Seymour *et al.*^28^ reported that higher proportion of patients achieved a SVC_O2_>70% within six hours when EMS initiated fluid therapy prior to arriving at the ED, the unadjusted risk ratio found no evidence of a difference (EMS IV fluids: 13/24 (54%) vs no IV fluids: 9/25 (36%), Unadjusted RR 1.5, 95% CI [0.8–2.9]). This same study also identified no improvement in time to MAP>65mmHg (EMS IV fluids: 17/24 (70%) vs no IV fluids: 12/26 (44%), unadjusted RR 1.53 (95% CI [0.9–2.65]), and time to CVP>8 mmH_2_0 (EMS IV fluids: 15/25 (60%) vs no IV fluids: 17/24 (70%), unadjusted RR 1.2 (95% CI [0.8–1.8]).^28^

Studnek *et al.*^5^ reported that if patients arrived by EMS they had shorter times to EGDT than if they arrived by other means (EMS: 119 minutes vs non-EMS: 160 minutes, SD/range not reported, p=0.005). Furthermore, among EMS-transported patients, if EMS documented suspicion of sepsis then time to EGDT was shorter than if they did not document suspicion of sepsis (documented suspicion: 69 minutes vs not documented: 131 minutes, SD/range not reported, p=0.001). McClelland *et al.*^25^ similarly reported that time to delivery of the ’Sepsis 6’ (administration of supplemental oxygen, intravenous fluids, antibiotics, measurement of venous lactate, urine output, and drawing blood to identify causative pathogen) was shorter if EMS identified sepsis prior to arrival at hospital (EMS identified: mean 205 minutes [SD 271 minutes, range 10–720 minutes] vs not identified: mean 120 minutes [SD 110, 17–450 minutes]). These data points include one outlier where the fluid balance chart was not started for 12 hours. Excluding this case, the mean time to delivery of the ‘Sepsis 6’ would be 76 minutes (SD 95 minutes, range 10–240 minutes).

## DISCUSSION

Very few, if any, EMS systems are capable of delivering the entire initial resuscitation bundle advocated by the Surviving Sepsis Campaign guidelines.^1^ Most EMS systems lack the capability to draw blood and analyze the required parameters; in addition some of the technical skills required, such as central line placement, will be beyond the scope of many non-physician providers. It is therefore unreasonable to expect EMS systems to be able to deliver all elements of the initial resuscitation bundle. However, key interventions, such as oxygen therapy, antibiotic administration, fluid resuscitation and measuring venous lactate are possible. Despite the ability of EMS to deliver the aforementioned, recent hospital trials^32^–^34^ have brought into question several of the EGDT objectives. We therefore need to examine carefully the need to extend EMS scope of practice to deliver those elements not routinely practiced, such as measuring venous lactate and administering antibiotics.

Prehospital recognition of sepsis is challenging.^8^,^27^,^35^ The limited evidence identified suggests the initiation of treatment by EMS may lead to improved process outcomes, i.e. reduces time taken to achieve initial resuscitation targets but is not necessarily associated with improved clinical outcomes.

There is currently no evidence addressing impact of prehospital oxygen therapy in sepsis. The ARISE^33^, ProCESS^32^ and ProMISe^34^ trials have all suggested that the need to rigidly adhere to EGDT may be overstated. Furthermore, a systematic review by Sterling *et al.*^36^ indicates that antibiotic administration within the first three hours is not associated with improved patient outcomes.

One study^29^ identified during this review suggests that prehospital antibiotics may reduce mortality (OR 0.56 (95% CI [0.32–1.00]), p=0.049); however, this study was published in abstract only and enrolled a limited number of patients (n=198). We cannot therefore be confident that prehospital antibiotics would improve outcomes. The PHANTASi trial (NCT01988428) will hopefully provide further evidence to determine if EMS systems should extend clinical practice to deliver prehospital antibiotic therapy in cases of suspected sepsis.

Fluid therapy is an established clinical practice in many EMS systems. Seymour *et al.*^31^ identified that prehospital fluid therapy was associated with both reduced organ failures (OR 0.58, 95% CI [0.34–0.98]) and mortality (OR 0.46, 95% CI [0.23–0.88]); however, the mean volume of fluid administered was only 500ml, considerably below what would normally be administered as part of the initial resuscitation bundle (30mL/kg).^1^ This led the authors to question if the reduced mortality was due to the small volume of fluid or indeed if it was associated with process improvements secondary to prehospital recognition of sepsis. The latter argument is strengthened by their finding that placement of an intravenous catheter, without any fluid being administered, was also associated with reduced hospital mortality (OR 0.31, 95% CI [0.17–0.57]).^31^

One further aspect that has not been examined is the influence of EMS system design. Internationally, two distinct EMS systems, the EMT/paramedic (Anglo-American) model and physician (Franco-German) model are observed. Typically physician responders might be expected to have higher clinical acumen than paramedics/EMTs as a result of their longer, more in-depth education and training. In addition they may have greater scope to initiate a broader range of interventions, as well as direct admission to specialist services. These factors could improve recognition and indeed treatment of sepsis before arriving at hospital.

Eight of the included studies were conducted in EMT/paramedic EMS systems^5^,^11^,^25^–^29^,^31^ with a single study, published in abstract only, conducted in a physician-based EMS system.^30^ Studies conducted in both system designs suggested reduced times to interventions; however, Bayer *et al.*^30^ did not publish data addressing mortality, ICU admission nor length of stay in their EMS physician-based study. Although Bayer *et al.*^30^ reported a high proportion of suspected prehospital sepsis cases were later confirmed in the hospital, they did not report data concerning missed cases making it impossible to determine if EMS physicians are able to accurately identify sepsis patients out of the hospital. Bayer *et al.*^30^ did however report a larger mean fluid volume (2.5l intravascular fluid (IQR 1.5–3.0l)),^30^ than in the paramedic-based study (mean volume 500mL (IQR 200–1000mL)) reporting this outcome,^31^ which may reflect greater understanding of beneficial treatments. With such limited data it is not possible to draw any meaningful conclusions concerning the impact of EMS physicians on outcomes in sepsis.

## LIMITATIONS

We employed a broad search strategy in order to capture as much published literature as possible. Inclusion criteria were similarly not restrictive so as to include as much of the evidence base as possible. To the best of our knowledge, this is the first systematic review addressing the impact of prehospital interventions upon outcomes among sepsis patients. Despite using very broad search criteria, little robust evidence regarding the impact of prehospital care of sepsis patients was identified. The studies found employed disparate methodologies, exhibit significant heterogeneity, generally involve small numbers of patients (limiting the precision of reported results) and were invariably of very low quality. The conclusions that can be drawn from this systematic review are therefore limited and findings should be interpreted with caution.

## CONCLUSION

There is little robust evidence addressing the impact of prehospital interventions on outcomes in sepsis. That which is available is of very low quality and indicates that prehospital interventions have limited impact on outcomes in sepsis beyond improving process outcomes and expediting the patients passage through the emergency care pathway. Evidence indicating that prehospital antibiotic therapy and fluid resuscitation improve patient outcomes is lacking. Well-conducted studies addressing key clinical interventions, such as antibiotic administration and fluid resuscitation are required.

## Supplementary Information



## Figures and Tables

**Figure:**
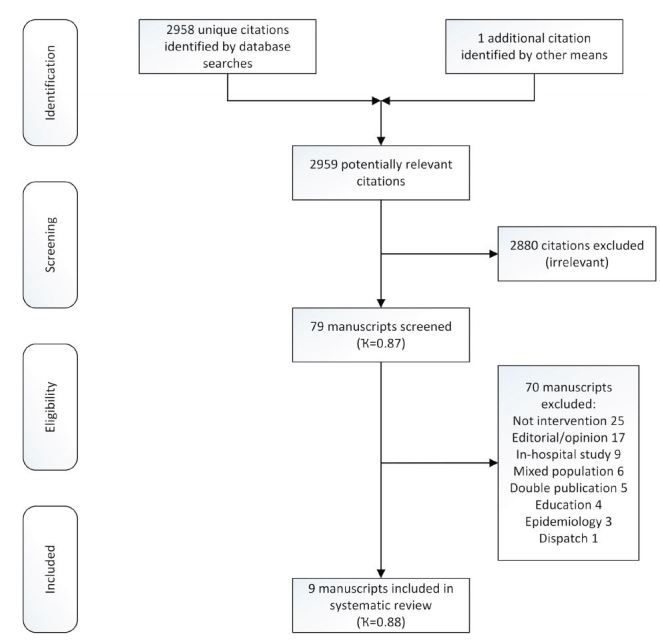
PRISMA flow chart. *PRISMA*, Preferred Reporting Items for Systematic Reviews and Meta-Analyses.

**Table 1:** Characteristics of studies reviewed for quality of evidence regarding whether early intervention by EMS prior to hospital arrival leads to improved clinical outcomes among sepsis patients.

Characteristic	Details
Median year of publication [range]	2013 [2009–2015]
Country of origin [n, (%)]	
Australia	1 (11)
Germany	1 (11)
United Kingdom	1 (11)
United States	6 (67)
Language [n, (%)]	
English	9 (100)
Study design [n, (%)]	
Randomized controlled trials	1 (11)
Non-randomized (observational) studies	8 (89)
Publication type	
Full publication	7 (78)
Abstract publication	2 (22)

*EMS,* emergency medical services.

**Table 2:**
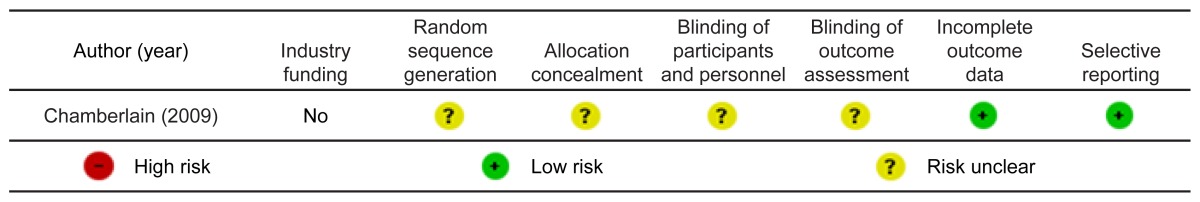
Risk of bias (randomized controlled trials).

**Table 3:**
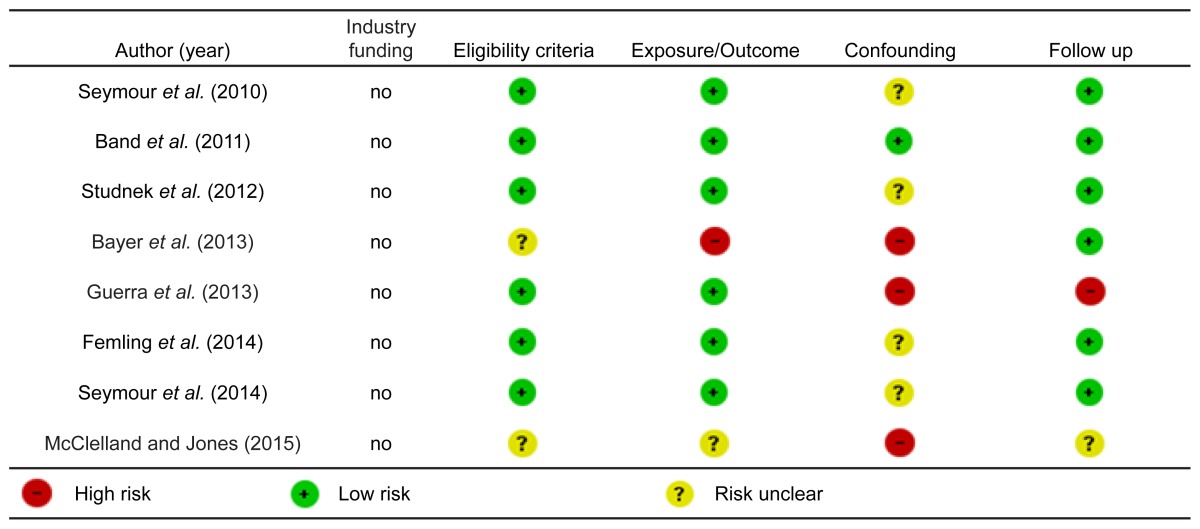
Risk of bias (non-randomized studies).

**Table 4:** Summary of findings.

No of studies	No of patients	Study design	Risk of bias	Inconsistency	Indirectness	Imprecision	Other	Level of evidence	Findings
Impact of prehospital care upon time to antimicrobial therapy									

1	199	RCT	not serious^1^	none	not serious^2^	very serious^3^	very serious^4^	⊕⊙⊙⊙ very low	[Chamberlain 2009] prehospital antibiotics provided 3.4 ± 2.6 hours sooner (p=0.02).
5	1,927	non-RCT	very serious^5^	none	not serious^2^	very serious^6^	very serious^7^	⊕⊙⊙⊙ very low	[Band 2011] Median time to antibiotics reduced: 116 minutes (IQR 66–199 minutes) EMS vs 152 minutes (IQR 92–252 minutes) ‘other means’ (p≤0.001). [Studnek 2012] if arriving by EMS vs other means time to antibiotics reduced 111 minutes (EMS) vs 146 minutes (non-EMS); (p=0.001). If EMS recognized and documented sepsis time to antibiotics reduced 70 minutes (documented) vs 122 minutes (not documented) (p=0.003). [Bayer 2013] Median time of administration 19 minutes (IQR 18–24 minutes) after initial emergency call (time of administration estimated as 10 minutes after arriving at scene). [Guerra 2013] No significant reduction in time to antibiotics mean 72.6 minutes (SD 59.3 minutes, pre-alert) vs 98.5 minutes (SD 89.9 minutes, no pre-alert) (p=0.07). [Femling 2014] Time to antibiotics: 87 minutes (EMS, IQR 44–157 minutes) vs 120 minutes (non-EMS, IQR 141–271 minutes), difference 33 minutes (p=0.02).

Impact of prehospital care upon fluid resuscitation									

5	2,697	non-RCT	very serious^5^	none	not serious^2^	very serious^6^	very serious^8^	⊕⊙⊙⊙ very low	[Seymour 2010] patients who received prehospital fluids had shorter time to MAP>65 mm Hg 17/24 (70%, EMS IV fluids) vs 12/26 (44%, no IV fluids), unadjusted RR 1.53 (95% CI [0.9–2.65]), and shorter time to CVP>8 mm H_2_0 15/25 (60%, EMS IV fluids) vs 17/24 (70%, no IV fluids), unadjusted RR 1.2 (95% CI [0.8–1.8]). [Band 2011] Median time to initiation of IVF reduced: 34 minutes (IQR 10–88) EMS vs 68 minutes (IQR 25–121 minutes) ‘other means’ of arrival (p≤0.001). [Bayer 2013] Median time of administration 19 minutes (IQR 18–24 minutes) after initial emergency call (time of administration estimated as 10 minutes after arriving at scene). Patients received 2.5L intravascular fluid (IQR 1.5–3.0L) until admitted to the ED. [Guerra 2013] No significant difference in fluid administration by 6 hours 42.97 cc/kg (SD 33.23cc/kg, pre-alert) vs 35.17cc/kg (SD 26.81 cc/kg, no pre-alert, p=0.30). [Seymour 2014] Median prehospital fluid volume 500mL (IQR 200–1000mL).

Impact of prehospital care upon Early Goal Directed Therapy									

6	2,523	non-RCT	very serious^5^	none	not serious^2^	very serious^6^	very serious^8^	⊕⊙⊙⊙ very low	[Seymour 2010] patients who received prehospital fluids had shorter time to MAP>65 mm Hg 17/24 (70%, EMS IV fluids) vs 12/26 (44%, no IV fluids), unadjusted RR 1.53 (95% CI [0.9–2.65]); shorter time to CVP>8 mm H_2_0 15/25 (60%, EMS IV fluids) vs 17/24 (70%, no IV fluids), unadjusted RR 1.2 (95% CI [0.8–1.8]); and shorter time to SVC_O2_>70% 13/24 (54%, EMS IV fluids) vs 9/25 (36%, no IV Fluids), unadjusted RR 1.5 (95% CI [0.8–2.9]). [Studnek 2012] if arriving by EMS vs other means time to EGDT reduced 119 minutes (EMS) vs 160 minutes (non-EMS, p=0.005). If EMS recognised and documented sepsis time to EGDT 69 minutes (documented) vs 131 minutes (not documented, p=0.001). [Guerra 2013] No significant reduction in proportion of patients with central venous line placement 62% (pre-alert) vs 68% (no pre-alert, p=0.54). [Femling 2014] Time to central line: 200 minutes (EMS, IQR 89–368 minutes) vs 275 minutes (non-EMS, IQR 122–470 minutes), difference 75 minutes (p<0.01). [Seymour 2014] Prehospital fluids reduced likelihood of increasing organ failures adjusted OR 0.58 (95% CI [0.34–0.98]). [McClelland 2015] Time to ‘sepsis 6’: mean 205 minutes (SD 271 minutes, range 10–720 minutes, EMS identified)* vs 120 minutes (SD 110, 17–450 minutes, not identified). (*Includes outlier where the fluid balance chart was not started for 12 hours, excluding this case mean 76 minutes [SD 95 minutes, range 10–240 minutes]).

Impact of prehospital care upon admission									

3	646	non-RCT	very serious^5^	none	not serious^2^	very serious^6^	very serious^8^	⊕⊙⊙⊙ very low	[Guerra 2013] No significant reduction in length of stay: mean 7.3 days (SD 6.8 days, pre-alert) vs 8.4 days (SD 8.8 days, no pre-alert, p=0.65). [Femling 2014] Length of stay: 15 days (IQR 13–17 days, EMS) vs 14 days (IQR 10–17 days, non-EMS), difference 1 day, not significant. [Seymour 2014] Prehospital vascular access reduced ICU admission adjusted OR 0.41 (95% CI [0.24 – 0.70]). [McClelland 2015] ICU admission: 4% (1/23, EMS identified) vs 13% (3/23, not identified).

Impact of prehospital care upon mortality									

5	2,959	non-RCT	very serious^5^	none	not serious^2^	very serious^6^	very serious^8^	⊕⊙⊙⊙ very low	[Band 2011] No significant difference in mortality was noted: adjusted RR 1.24 (95% CI [0.92 – 1.66, p=0.16). [Guerra 2013] If hospital was ‘pre-alerted’, unadjusted mortality was improved OR 3.19 (95% CI [1.14–8.88], p=0.04). [Femling 2014] No significant difference in mortality was noted 113/378 (30%, EMS) vs 34/107 (31%, non-EMS), difference 1%, not significant. [Seymour 2014] Prehospital vascular access reduced mortality adjusted OR 0.31 (95% CI [0.17 – 0.57], p<0.01). [McClelland 2015] 3 month mortality 21% (5/24, EMS identified) vs 16% (4/25, not identified).

Impact of prehospital antimicrobial therapy on ICU admission									

1	199	RCT	not serious^1^	none	not serious^2^	very serious^3^	very serious^4^	⊕⊙⊙⊙ very low	[Chamberlain 2009] Mean ICU length of stay: reduced 6.8 ± 2.1 days (intervention) vs 11.2 ± 5.2 days (control, p=0.001).

Impact of prehospital antimicrobial therapy on mortality									

1	199	RCT	not serious^1^	none	not serious^2^	very serious^3^	very serious^4^	⊕⊙⊙⊙ very low	[Chamberlain 2009] 28-day mortality reduced: 42.4% (intervention) vs 56.7% (control), OR 0.56 (95% CI [0.32 to 1.00], p=0.049).

Impact of prehospital intravenous fluid therapy on ICU admission									

1	1,350	non-RCT	not serious^9^	none	not serious^2^	none	none	⊕⊙⊙⊙very low	[Seymour 2014] Prehospital fluids did not reduce likelihood of ICU admission adjusted OR 0.64 (95% CI [0.37–1.10]).

Impact of prehospital intravenous fluid therapy on mortality									

1	1,350	non-RCT	not serious^9^	none	not serious^2^	none	none	⊕⊙⊙⊙very low	[Seymour 2014] Prehospital fluids reduced hospital mortality adjusted OR 0.46 (95% CI [0.23–0.88], p=0.02).
Risk of bias unclear. Single centre study may limit generalizability. Small study numbers limits precision/accuracy. Published in abstract only, insufficient detail to rule out other bias. Concerns relating to eligibility, exposure, confounding, follow-up Small study numbers limits precision/accuracy, failure to report confidence intervals (Guerra) Abstract only publication (Femling), insufficient detail to rule out other bias, Publication bias likely (Guerra) Publication bias likely (Guerra) Risk of bias unclear									

*RCT*, randomized control trial; *EMS*, emergency medical services; *ED*, emergency department; *IQR,* interquartile range; *CI,* confidence interval; *RR,* risk ratio; *MAP,* mean arterial pressure; *CVP,* central venous pressure; *IVF,* intravascular fluid; *IV,* intravascular; *SVC_O2_*, superior vena cava oxygen; *EGDT*, early goal directed therapy; *OR,* odds ratio; *ICU,* intensive care unit; *RCT,* non-randomized controlled trial (observational study); *SD,* standard deviation.
