# Supplementary material for: Impact of Prehospital Care on Outcomes in Sepsis: A Systematic Review
Source: West J Emerg Med. 2016 Jul 5;17(4):427–37. doi: 10.5811/westjem.2016.5.30172 (PMC4944799; doi:10.5811/westjem.2016.5.30172)
Supplement: Supplementary file 1 [file wjem-17-427-s001.docx]

*Appendix A: Impact of EMS care on outcomes in sepsis.*

| **Author (year),**  **Country** | **Study Design (n)** | **Publication Type** | **Study Population** | **Study Intervention** | **Key findings** | | | | **Quality Assessment/Limitations** | |
| --- | --- | --- | --- | --- | --- | --- | --- | --- | --- | --- |
| Chamberlain (2009), Australia | RCT  n=198 | abstract | Prehospital sepsis patients | Patients randomised to prehospital antimicrobial plus fluid therapy or fluid therapy alone. | Time to antimicrobial was reduced by 3.4 ± 2.6 hours (p=0.02)  Mean ITU length of stay shorter: 6.8 ± 2.1 days (intervention) vs 11.2 ± 5.2 days (control), (p=0.001)  28-day mortality reduced: 42.4% (intervention) vs 56.7% (control), OR 0.56 (95% CI 0.32 to 1.00; p=0.049) | | | | *Risk of bias* | Risk unclear (see bias assessment) |
|  |  |  |  |  |  |  |  |  | *Inconsistency* | None |
|  |  |  |  |  |  |  |  |  | *Indirectness* | Not serious: probable single centre study may limit generalisability |
|  |  |  |  |  |  |  |  |  | *Imprecision* | Very serious: small study numbers limits precision/accuracy |
|  |  |  |  |  |  |  |  |  | *Other* | Very Serious: published in abstract only |
| Seymour et al (2010b), USA | non-RCT n=216 | paper | Emergency Department sepsis patients who arrived by EMS | Does EMS IV fluid (IVF) improve time to EGDT: MAP>65mmHg, CVP>8cmH_2_O, ScvO_2_>70% | 76/216 patients had ED EGDT documented. 25/76 ALS IVF, 27/76 ALS no IVF (IV access failed 10/27), 20/76 BLS, 4/76 insufficient EMS data. | | | | *Risk of bias* | Risk unclear (see bias assessment) |
|  |  |  |  |  | *Variable* | *IVF* | *No IVF* | *Unadj RR (95% CI)* | *Inconsistency* | None |
|  |  |  |  |  | MAP>65mmHg | 17/24 (70%) | 12/26 (44%) | 1.53 (0.9-2.65) | *Indirectness* | Not serious: single centre study may limit generalisability |
|  |  |  |  |  | CVP>8cmH_2_O | 18/25 (72%) | 15/25 (60%) | 1.2 (0.8-1.8) | *Imprecision* | Very serious: small study numbers limits precision/accuracy |
|  |  |  |  |  | ScvO_2_>70% | 13/24 (54%) | 9/25 (36%) | 1.5 (0.8- 2.9) | *Other* | None |
| Band et al (2011), USA | non-RCT n=963 | paper | Emergency Department sepsis patients | Comparison of time to antibiotics, time to intravascular fluid (IVF) therapy and mortality among ED patients arriving by EMS vs ‘other means’. | Median time to antibiotics reduced: 116 min (IQR 66-199min) EMS vs 152 min (IQR 92-252min) ‘other means’, (p≤0.001).  Median time to initiation of IVF reduced: 34 min (IQR 10 - 88) EMS vs 68 min (IQR 25-121min) ‘other means’, (p≤0.001).  No significant difference in mortality was noted: adjusted RR 1.24 (95% CI 0.92 - 1.66), (p=0.16). | | | | *Risk of bias* | Low risk (see bias assessment) |
|  |  |  |  |  |  |  |  |  | *Inconsistency* | None |
|  |  |  |  |  |  |  |  |  | *Indirectness* | Not serious: single centre study may limit generalisability |
|  |  |  |  |  |  |  |  |  | *Imprecision* | None |
|  |  |  |  |  |  |  |  |  | *Other* | None |
| Studnek et al (2102), USA | non-RCT n=311 | paper | Emergency Department sepsis patients | Comparison of SOFA score, time to antibiotics, time to EGDT among ED patients arriving by EMS vs ‘other means’. | 160/311 patients transported by EMS  SOFA scores 7.0(EMS) vs 6.1(non-EMS); (p=0.02)  Time to antibiotics 111(EMS) vs 146min(non-EMS); (p=0.001)  Time to EGDT 119(EMS) vs 160 min(non-EMS); (p=0.005)  If EMS documented suspected sepsis  Time to antibiotics 70(doc) vs 122min(no doc); (p=0.003)  Time to EGDT 69(doc) vs 131 min(no doc); (p=0.001) | | | | *Risk of bias* | Risk unclear (see bias assessment) |
|  |  |  |  |  |  |  |  |  | *Inconsistency* | None |
|  |  |  |  |  |  |  |  |  | *Indirectness* | Not serious: single centre study may limit generalisability |
|  |  |  |  |  |  |  |  |  | *Imprecision* | Very serious: small study numbers limits precision/accuracy, failed to report confidence intervals |
|  |  |  |  |  |  |  |  |  | *Other* | None |
| Bayer et al (2013), Germany | non-RCT n=56 | abstract | Prehospital sepsis patients | Reduction in time to antibiotics, and time to intravascular fluids among patients managed by EMS physician. | Aribitrary estimation of time to antibiotic and fluid administration of 10 minutes post arrival on scene.  Median time of administration calculated as 19min (IQR 18-24min) after initial emergency call.  Arrived at ED median 56min (IQR 46-67min), however not clear if this time is calculated from initial call or following arrival of EMS physician at scene.  Patients received 2.5l (IQR 1.5–3.0l) intravascular until admitted to the ER. | | | | *Risk of bias* | High risk (see bias assessment) |
|  |  |  |  |  |  |  |  |  | *Inconsistency* | None |
|  |  |  |  |  |  |  |  |  | *Indirectness* | Not serious: physician based EMS, may not be generalisable |
|  |  |  |  |  |  |  |  |  | *Imprecision* | Very Serious: small study numbers limits precision/accuracy |
|  |  |  |  |  |  |  |  |  | *Other* | Very Serious: published in abstract only |
| Guerra et al (2013), USA | non-RCT n=112 | paper | Sepsis patients admitted to hospital who arrived by EMS | Comparison of patient characteristics and key outcomes among sepsis patients recognised by EMS vs those not recognised by EMS | If hospital was ‘pre-alerted’, survival to discharge improved OR 3.19 (95% CI 1.14– 8.88; p=0.040);  No significant reduction in time to antibiotics mean 72.6min (SD 59.3min) (pre-alert) vs 98.5min (SD 89.9min) (no pre-alert), p=0.07  No significant reduction in length of stay mean 7.3 days (SD 6.8days) (Pre-alert) vs 8.4 days (SD 8.8 days) (no pre-alert), p=0.65.  No significant reduction in proportion of patients with central venous line placement 61% (pre-alert) vs 68% (no pre-alert) p=0.54  No significant difference in fluid administration by 6 hours 42.97 cc/kg (SD 33.23cc/kg) (pre-alert) vs 35.17cc/kg (SD 26.81 cc/kg) (no pre-alert), p=0.30. | | | | *Risk of bias* | High risk (see bias assessment) |
|  |  |  |  |  |  |  |  |  | *Inconsistency* | None |
|  |  |  |  |  |  |  |  |  | *Indirectness* | Not serious: lactate not widely available within EMS |
|  |  |  |  |  |  |  |  |  | *Imprecision* | Very Serious: small study numbers limits precision/accuracy, failed to report confidence intervals. |
|  |  |  |  |  |  |  |  |  | *Other* | Very Serious: publication bias probable |
| Femling et al (2014) | non-RCT  n=485 | paper | Emergency Department sepsis patients | Comparison of patient characteristics plus time to antibiotics, time to central line, ITU stay and mortality among ED patients arriving by EMS vs ‘other means’. | Time to antibiotics: 87min (EMS) (IQR 44-157min) vs 120min (non-EMS) (IQR 141-271min), diff 33min, p=0.02.  Time to central line: 200min (EMS) (IQR 89-368min) vs 275min (non-EMS) (IQR 122-470min), diff 75min, p<0.01.  Length of stay: 15 days (EMS) (IQR13-17days) vs 14days (IQR 10-17days), diff 1 day, not significant.  Mortality: 113/378 (30%) (EMS) vs 34/107 (31%) (non-EMS), diff 1%, not significant. | | | | *Risk of bias* | Risk unclear (see bias assessment) |
|  |  |  |  |  |  |  |  |  | *Inconsistency* | None |
|  |  |  |  |  |  |  |  |  | *Indirectness* | Not serious: single centre study may limit generalisability |
|  |  |  |  |  |  |  |  |  | *Imprecision* | None |
|  |  |  |  |  |  |  |  |  | *Other* | None |
| Seymour et al (2014), USA | non-RCT n=1350 | paper | Severe sepsis patients admitted to hospital who arrived by EMS | Risk-adjusted association between prehospital  fluid administration and hospital mortality. | 201/1350 died before discharge. 948/1350 no vascular access, 90/1350 IV access no fluid, 312/1350 IV fluids.  Prehospital vascular access reduced mortality OR 0.3 (95% CI 0.17 - 0.57), (p<0.01).  Prehospital fluids reduced hospital mortality OR 0.46 (95% CI 0.23-0.88), (p=0.02).  Median prehospital fluid volume 500mL (IQR 200-1000mL).  Prehospital fluids reduced likelihood of increasing organ failures OR 0.58 (95% CI 0.34-0.98) but did not reduce likelihood of ITU admission OR 0.64 (95% CI 0.37-1.10) | | | | *Risk of bias* | Risk unclear (see bias assessment) |
|  |  |  |  |  |  |  |  |  | *Inconsistency* | None |
|  |  |  |  |  |  |  |  |  | *Indirectness* | Not serious: single centre study may limit generalisability |
|  |  |  |  |  |  |  |  |  | *Imprecision* | None |
|  |  |  |  |  |  |  |  |  | *Other* | None |
| McClelland and Jones (2015), UK | non-RCT n=49 | paper | Sepsis patients admitted to hospital who arrived by EMS | Comparison of patient characteristics and key outcomes among sepsis patients recognised by EMS vs those not recognised by EMS | EMS identified sepsis;  ICU admission 4% (1/23) (EMS identified) vs 13% (3/23) (not identified)  3 month mortality 21% (5/24) (EMS identified) vs 16% (4/25) (not identified)  Time to sepsis 6 mean 205min (SD 271min, range 10-720min)* (EMS identified) vs 120 min (SD 110, 17-450min) (not identified) (* includes outlier where the fluid balance chart was not started for 12 hours, excluded this case mean 76min (SD 95min, range 10-240min)) | | | | *Risk of bias* | High risk (see bias assessment) |
|  |  |  |  |  |  |  |  |  | *Inconsistency* | None |
|  |  |  |  |  |  |  |  |  | *Indirectness* | Not serious: single centre study may limit generalisability |
|  |  |  |  |  |  |  |  |  | *Imprecision* | Very Serious: small study numbers limits precision/accuracy |
|  |  |  |  |  |  |  |  |  | *Other* | None |
